# Adolescent risk factors for purging in young women: findings from the national longitudinal study of adolescent health

**DOI:** 10.1186/2050-2974-2-1

**Published:** 2014-01-03

**Authors:** Eric M Stephen, Jennifer Rose, Lindsay Kenney, Francine Rosselli-Navarra, Ruth Striegel Weissman

**Affiliations:** 1 Department of Psychology, Wesleyan University, 207 High Street, Middletown, CT 06459, USA; 2 Department of Psychology, Manchester Community College, Great Path MS#4, Manchester, CT, USA

**Keywords:** Eating disorders, Risk factors, Purging behavior, Weight control, Adolescent

## Abstract

**Background:**

There exists a dearth of prospective adolescent eating disorder studies with samples that are large enough to detect small or medium sized effects for risk factors, that are generalizable to the broader population, and that follow adolescents long enough to fully capture the period of development when the risk of eating disorder symptoms occurring is highest. As a result, the purpose of this study was to examine psychosocial risk factors for purging for weight control in a nationally representative sample of adolescents. Data were extracted from the restricted-use data sets of the National Longitudinal Study of Adolescent Health (Waves I-III), selecting females with valid demographic and purging information (N = 5,670).

**Results:**

The prevalence of purging was 0.88% at Wave II and 0.56% at Wave III. In multivariable multinomial logistic regressions, purging at Wave II was predicted by parental poverty and low levels of self-esteem at Wave I; purging at Wave III was predicted by body mass index and the frequency of delinquent behaviors at Wave I.

**Conclusions:**

Individuals with high body mass index, individuals with low self-esteem, and individuals in families experiencing economic hardship appear specifically at risk for the development of purging behaviors in later years and may benefit from more targeted prevention efforts.

## Background

The etiologies of eating disorders and their core symptoms are best characterized by multifactorial models involving biological, psychological, interpersonal, and cultural variables [1]. Experts have also noted, however, that the research is limited by the dearth of prospective studies with samples large enough to detect small or medium sized effects, the inclusion of demographically limited samples, and the lack of follow-up periods that are long enough to fully capture the period of development when the risk of the onset of eating disorder symptoms is highest [1,2].

The present study focused on purging (vomiting or abusing laxatives) to lose weight or keep from gaining weight as a clinically relevant symptom and as a possible proxy for the presence of an eating disorder. Purging behaviors have received increasing scientific attention not only in the context of a full syndrome eating disorder but also as symptoms of clinical relevance in their own right [3-5]. Only a few studies have explored risk factors for purging using longitudinal designs, and most of these studies have focused on just one risk factor. For example, two studies of adolescent girls found that eating dinner [6] or eating meals with their families [7] was associated with a decreased risk for onset of purging or extreme weight control behaviors.

Due to skip patterns used in most epidemiological studies of adults, information about purging behaviors typically was collected only in individuals who scored positively on the core symptom of anorexia nervosa (refusal to maintain a minimum adequate body weight) or bulimia nervosa (recurrent episodes of purging and binge eating) [8]. In contrast, studies of adolescents typically have measured disordered eating symptoms among all participants, thus affording the opportunity to explore correlates of these behaviors. Results show marked gender differences in the incidence and prevalence of purging [9]. For example, in a community sample of over 13,000 boys and girls (ages 9 to 14 years), one-year incidence of purging was 1.3% in girls and 0.3% in boys [6]. In a school-based sample of over 2,500 boys and girls, “extreme weight control behaviors” (which included purging) were also reported to be more common in girls than boys [10]. The present study capitalized upon the availability of data collected as part of the National Longitudinal Study of Adolescent Health (Add Health), which affords the unique opportunity to examine the prevalence and incidence of, as well as risk factors for, purging in a nationally representative sample of United States’ (US) youth. Because of the low base rate of males who purged in our sample (only 0.55% of young men reported purging at any wave of study whereas 1.96% of young women reported purging), we excluded male participants from our sample.

Our approach to and selection of potential risk factors was informed by Kraemer and colleagues’ conceptual framework and by a corresponding review paper of the empirical studies of risk factors for eating disorders [2,11]. Specifically, to test whether a variable confers risk (rather than merely representing a correlate), data are needed that were either collected prior to occurrence of the behavior or are of a nature where one may reasonably infer that, even when collected after occurrence, the data are not contaminated by the outcome in question (e.g., gender, race/ethnicity). Therefore, in our study, we defined occurrence, or “incidence cases”, as participants who reported purging at a subsequent wave of study, controlling for purging behaviors at Wave I. Given our interest in identifying adolescent risk factors for purging, we limited our sample to adolescent girls who, at study entry (Wave I), were age 18 or younger.

Our study included a considerable range of potential adolescent risk factors including demographic variables, early onset menarche, body image concerns, adverse childhood experiences, coping, and health or mental health problems [2]. We hypothesized that, after controlling for age, race, parental education, and purging at Wave I, higher scores on each of these risk factor constructs (and, in the case of self-esteem, lower scores) would confer greater risk for the occurrence of purging behaviors in young adult women.

## Methods

### Participants

The sample comprised 5,670 female participants whose data were extracted from the restricted-use Add Health survey Waves I (1994–1995), II (1996), and III (2001–2002). Add Health has been following over 20,000 American adolescents starting in grades 7–12 over the course of fourteen years, with four waves of data collection throughout this time period (Wave IV did not collect data about purging and, therefore, was not used for the present study). The initial sample was established by sampling students from 132 schools in the United States. Due to oversampling of some population subgroups, Add Health investigators developed sampling weights to be used in statistical analyses so findings can be generalized to be representative for region, urbanicity, and ethnicity. Further details about the Add Health study design can be found elsewhere [12,13].

Inclusion criteria for the present study were those participants who were reported as female at all three waves (in a few instances, gender information was not consistent across study waves), had valid age information, were age 18 or younger at Wave I, reported responses to purging behaviors at Wave I, and had valid data required for applying sampling weights (see Data Analysis section). Of the total 10,480 girls included in Add Health at Wave I, 5,670 met the inclusion criteria required for our analyses (54% Wave I sample, 75% of Wave II sample, and 71% of Wave III sample). Most of the girls that we excluded did not have information needed for applying sample weights at all waves. However, because we used the sample weights developed by Add Health in our statistical models to account for attrition and oversampling, our findings are representative of young women in the United States.

The mean age at study entry was 15.15 (SD = 1.57); about one-third (30.97%) of the weighted sample was non-White. Highest parental education was reported by participants’ parents or guardians as follows: less than high school, 11.65%; high school diploma or equivalent, 32.10%; some college, 22.38%; and college degree or more, 33.87%. All results reflect adjustment for sampling strategy [13,14].

### Instruments and procedures

The Add Health survey measures a wide range of psychological and physical health issues and behaviors, a subset of which was used in this study. Some constructs (e.g., depressive symptoms) were measured using unequal numbers of items across waves; in such cases, we followed the practice of prior investigators of using only those items that were administered in all three waves, and we calculated Cronbach’s alpha based on the unweighted sample included in the present study.

Because, by definition, exposure to a risk factor must occur prior to occurrence of the dependent variable of interest, predictor variables collected at Wave I were used to investigate purging at Wave II and Wave III. Attention-deficit hyperactivity symptoms and childhood abuse were the only predictor variables used in this study that were collected at Wave III, as they were measured retrospectively at Wave III with a focus on experiencing them in early childhood.

### Demographic information

Following work by Brown and colleagues, [15] participants’ self-reported race/ethnicity was coded into a binary variable representing White vs. non-White, and highest parental education achieved by either parent or adult guardian was coded as an ordinal variable representing less than high school, high school or equivalent, some college, and college graduate or postgraduate education. Age was recorded as age at last birthday. (Age at Wave I needed to be calculated because only birth year and birth month were available in Wave I. To calculate age, information given on birth date was subtracted from date of interview).

### Body image and eating behaviors

Body Mass Index (BMI) was calculated by dividing weight (in pounds) by the product of height (in inches) squared times the value 703 [16]. Weight perception was measured using a single item “How do you think of yourself in terms of weight?” with response choices ranging from “1 = very underweight” to “5 = very overweight”. Purging status was determined based on participants’ responses to the question of whether, in the past 7 days, the participant had been trying to lose or maintain weight. If the respondent answered yes, Add Health asked a series of follow up questions to determine what method the individuals were using to lose or maintain weight. Participants could select multiple options that were provided, including diet, exercise, vomiting, diet pill use, laxative use, or other. Those who reported that they had, in the last 7 days, tried to lose or maintain weight through the use of vomiting and/or laxative misuse were coded as purging. Questions did not address frequency of occurrence. Diet pill use in the absence of purging was coded as present if respondents reported use of diet pills but denied vomiting or laxative use.

### Classification of participants as current purgers

A three-level polyotomous variable was created to classify current purging. The first group was comprised of participants who did not report purging at either Wave II or Wave III. The second group was comprised of those reported purging at Wave II. Finally, all individuals who did not report purging at Wave II but did report purging at Wave III were coded into the third group. A small number of individuals reported purging at both Waves II and III (0.04% of the weighted sample), and were placed into the Wave II group.

### Reproductive health related variables

Early onset menarche was coded as present in girls reporting having their first menstrual period before age 11 years [17]. Pregnancy was coded as present if the respondent reported having been pregnant, including any pregnancy that ended in abortion, stillbirth, miscarriage, or live birth after which the baby died.

### Self-esteem

Four questions based on Rosenberg’s Self-Esteem Scale [18] were used to measure self-esteem, including “You have many good qualities”, “You have much to be proud of”, “You like yourself just the way you are”, and “You feel you are doing things just right”. Participants rated each item on a scale from 1 to 5, and, consistent with Kort-Butler et al., [19] a total score was calculated by adding across the items, where higher numbers indicate higher self-esteem. (Cronbach’s alpha = 0.80).

### Active coping

Four questions were used to measure active coping, including “When you have a problem to solve, the first thing you do is get as many facts about the problem as possible”, “When you are attempting to find a solution to a problem, you usually try to think of as many different ways to approach the problem as possible”, “When making decisions, you generally use systematic methods for judging and comparing alternatives”, and “After carrying out a solution to a problem, you usually try to analyze what went right and what went wrong”. Each item was rated on a scale from 1 to 5; a total score was created by summing across the items, and higher scores reflected more active coping. [15] (Cronbach’s alpha = 0.75).

### Mental health related variables

Depressive symptoms were measured using a modified version of the Center for Epidemiologic Studies-Depression (CES-D) scale [20]. Participants rated symptom frequency in the past week on a scale from 0 (never or rarely) to 3 (most of the time). In the present study we used the nine items that were asked at all three waves, and created a total score for Wave I. [21] (Cronbach’s alpha = 0.81). In addition, participants were asked, “During the last 12 months, how many times did you actually attempt suicide?” A binary variable (attempted suicide) was created contrasting those who had not attempted suicide during the past 12 months with those who had attempted suicide one or more times. Participants were asked (no/yes) whether, in the past 12 months, they had “received psychological or emotional counseling” (psychological counseling). Attention-deficit/hyperactivity Disorder (ADHD) was measured in Wave III. Participants were asked to “Think back to when you were between 5 and 12 years of age. For each of the following statements, which answer best describes your behavior when you were that age?” A list of all but one of the symptoms for ADHD as described in the 4^th^ edition of the Diagnostic and Statistical Manual for Mental Disorders (DSM-IV) [22] was then presented, and participants rated how frequently the symptoms were experienced (“never or rarely”, “sometimes”, “often”, or “very often”). The impulsivity symptom “often interrupts or intrudes on others” was not measured in Add Health. Consistent with McClernon and colleagues, [23] each symptom was coded as present if the frequency rating was “often” or “very often”. Two severity subscales were created by summing the nine binary inattention symptoms (ADHD-IN; Cronbach’s alpha = 0. 80) and the eight binary hyperactivity-impulsivity items (ADHD-HI; Cronbach’s alpha = 0.72).

### Delinquency

Seven questions measuring delinquent behavior were available at each wave. Specifically, participants were asked “In the past 12 months, how often did you: (1) damage property that didn’t belong to you; (2) steal something worth more than $50; (3) go into a house or building to steal something; (4) use or threaten to use a weapon to get something from someone; (5) sell marijuana or other drugs; (6) steal something worth less than $50; (7) take part in a fight where a group of your friends was against another group”. Consistent with previous studies, responses to each individual question were dichotomized by recoding the original 4-point Likert format (never, one or two times, three or four times, five times or more) into never versus ≥ 1. The recoded items were then summed, generating a Delinquency Scale total score with a range from 0 to 7. [24] The reliability of this scale was modest (Cronbach’s alpha = 0.61).

### Childhood adversity

Family poverty was measured using a parent’s responses to three questions (no = 0, yes = 1): receiving public assistance, aid to families with dependent children, or food stamps; responses were summed for a total family poverty score with higher scores indicating greater economic hardship (Cronbach’s alpha = 0.86). Binary variables were created indicating whether the participant had experienced these events for at least one parent: never lived with parent and parental death. In Wave III, participants were asked to retrospectively report (no/yes) whether, before the 6th grade, they had experienced childhood abuse (from parents or other adult caretakers): “not taking care of your basic needs, such as keeping you clean or providing food and clothing?” (childhood neglect) or “slapped, hit or kicked you?” (childhood physical abuse).

### Data analysis

All data analyses were performed using Stata 12.1 [24]. As part of Add Health’s survey design, certain demographic subgroups were sampled with unequal probability. In order to generate nationally representative estimates, therefore, Add Health investigators developed a weighting system which includes a stratum variable to adjust for geographic stratification, a cluster variable to adjust for the primary sampling unit (PSU; i.e. the school identifier), and a weight variable (using Wave III weights) to adjust for the oversampling of certain demographic characteristics within each PSU [13,25]. All analyses and estimates were performed using the weighted sample.

For each hypothesized risk or protective factor, multinomial logistic regression analysis was conducted, adjusting for the potentially confounding effects of age, race, parental education, and purging at Wave I. In the next step, a multivariable multinomial regression model was tested including only those risk or protective factors that had been shown to significantly differ (p < 0.05) between any two groups within the univariable comparisons.

## Results

### Prevalence and incidence of purging across the three waves

Unweighted frequencies and weighted proportions for purging are provided in Table 1. Regarding the weighted sample, the prevalence of purging was 0.40% at Wave I. The prevalence of current purging, as it was defined above, was 0.88% at Wave II and 0.56% at Wave III.

**Table 1:** Sample demographic characteristics (n = 5670)

**Variable**	**N**	**Unweighted %**	**Weighted %**	
Purging occurrence, Wave II	58	0.54%	0.88%	
Purging occurrence, Wave III	36	0.33%	0.56%	
Race Other Than White	2507	44.22%	30.97%	
Education				
Less than HS	689	12.15%	11.65%	
HS diploma	1625	28.66%	32.10%	
Some college	1155	20.37%	22.38%	
College diploma	1953	34.44%	33.87%	
	Unweighted mean	Unweighted SD		
Age^1^	15.15	1.57		

^1^Weighted descriptives unavailable for quantitative variables.

### Risk factors for the development of current purging

As shown in Table 2, among the variables pertaining to body image and eating behavior, overweight self-perception was found to be significantly associated with purging at Wave II: a significantly higher percentage of young women with purging at Wave II reported that they felt overweight at Wave I compared to those who were not purging at Wave II. Purgers at Waves II and III had significantly higher BMI at Wave I. However, BMI at Wave I did not predict differences in the likelihood of purging between Waves II and III. Using diet pills as weight control was not associated with purging at Waves II or III, nor was early-onset menarche or teenage pregnancy.

**Table 2:** Association of risk and protective factors with likelihood of purging at Waves II and III

**Variable**	**Purging behavior**	**Coefficient**	**Std. error**	**p-value**	**CI**
Weight perception (Likert)	Wave II	**1.01**	**0.37**	**0.01**	**0.28, 1.74**
	Wave III	0.54	0.49	0.27	-0.42, 1.50
	Wave III - Wave II	-0.47	0.60	0.44	-1.65, 0.72
Body mass index	Wave II	**0.09**	**0.03**	**0.00**	**0.03, 0.14**
	Wave III	**0.09**	**0.04**	**0.02**	**0.01, 0.16**
	Wave III - Wave II	0.00	0.05	0.99	-0.09, 0.09
Diet pill use	Wave II	1.16	0.97	0.24	-0.76, 3.08
	Wave III	1.24	1.06	0.25	-0.86, 3.34
	Wave III - Wave II	0.08	1.40	0.95	-2.68, 2.85
Menses onset < age 11 years	Wave II	0.37	0.47	0.43	-0.56, 1.29
	Wave III	0.13	0.74	0.87	-1.34, 1.60
	Wave III - Wave II	-0.24	0.89	0.79	-1.99, 1.52
Teenage pregnancy	Wave II	-0.83	0.94	0.38	-2.69, 1.04
	Wave III	-0.41	0.95	0.67	-2.28, 1.47
	Wave III - Wave II	0.42	1.34	0.75	-2.22, 3.06
Self-esteem scale	Wave II	**-0.22**	**0.06**	**0.00**	**-0.33, -0.11**
	Wave III	-0.01	0.09	0.90	-0.20, 0.17
	Wave III - Wave II	**0.21**	**0.10**	**0.04**	**0.00, 0.42**
Depressive symptoms scale	Wave II	**0.12**	**0.03**	**0.00**	**0.07, 0.18**
	Wave III	**0.09**	**0.03**	**0.01**	**0.02, 0.15**
	Wave III - Wave II	-0.04	0.04	0.31	-0.12, 0.04
Attempted suicide	Wave II	**1.14**	**0.47**	**0.02**	**0.21, 2.07**
	Wave III	0.25	0.66	0.71	-1.05, 1.54
	Wave III - Wave II	-0.90	0.81	0.27	-2.49, 0.70
Had psychological counseling	Wave II	**1.05**	**0.36**	**0.01**	**0.33, 1.77**
	Wave III	0.25	0.66	0.70	-1.05, 1.56
	Wave III - Wave II	-0.79	0.78	0.31	-2.34, 0.75
Delinquency scale	Wave II	**0.25**	**0.10**	**0.02**	**0.04, 0.45**
	Wave III	-0.32	0.28	0.26	-0.88, 0.24
	Wave III - Wave II	-0.57	0.29	0.06	-1.15, 0.01
Inattentiveness scale^3^	Wave II	0.11	0.09	0.23	-0.07, 0.29
	Wave III	0.03	0.14	0.84	-0.25, 0.30
	Wave III - Wave II	-0.08	0.18	0.65	-0.43, 0.27
Hyperactive/impulsive scale^3^	Wave II	**0.15**	**0.06**	**0.02**	**0.03, 0.26**
	Wave III	-0.04	0.15	0.80	-0.34, 0.27
	Wave III - Wave II	-0.18	0.17	0.29	-0.53, 0.16
Active coping scale	Wave II	-0.05	0.06	0.41	-0.18, 0.07
	Wave III	-0.08	0.07	0.25	-0.21, 0.06
	Wave III - Wave II	-0.03	0.09	0.77	-0.21, 0.15
Never lived with parent (s)	Wave II	**1.42**	**0.53**	**0.01**	**0.37, 2.46**
	Wave III	0.38	0.77	0.63	-1.15, 1.91
	Wave III - Wave II	-1.04	0.93	0.27	-2.88, 0.81
Parental poverty	Wave II	**0.42**	**0.19**	**0.03**	**0.05, 0.78**
	Wave III	-0.14	0.43	0.75	-1.00, 0.71
	Wave III - Wave II	-0.56	0.40	0.17	-1.35, 0.24
Not eating with parent	Wave II	-0.21	0.40	0.60	-1.00, 0.58
	Wave III	0.35	0.49	0.47	-0.61, 1.31
	Wave III - Wave II	0.56	0.66	0.40	-0.75, 1.87
Parent died	Wave II	-0.18	0.64	0.78	-1.44, 1.08
	Wave III	0.49	0.74	0.51	-0.97, 1.95
	Wave III - Wave II	0.66	0.95	0.49	-1.22, 2.54
Childhood neglect^3^	Wave II	0.46	0.56	0.41	-0.64, 1.56
	Wave III	-0.62	0.84	0.47	-2.29, 1.05
	Wave III - Wave II	-1.08	1.02	0.29	-3.10, 0.94
Childhood physical abuse^3^	Wave II	-0.42	0.44	0.35	-1.29, 0.45
	Wave III	0.64	0.51	0.21	-0.37, 1.65
	Wave III - Wave II	1.06	0.71	0.14	-0.35, 2.46

Note: All risk factor variables were measured at Wave I except those marked ^3^; the latter were measured at Wave III via self-reported retrospective recall. Those reported in bold are significant at p<0.05.

Several significant effects were found in the mental health domain. Those who purged at Wave II had lower levels of self-esteem at Wave I; interestingly, those who purged at Wave III had higher levels of self-esteem at Wave I than those who purged at Wave II, but self-esteem was not associated with purging at Wave III. Higher scores on the depressive symptom scale at Wave I were significantly associated with purging at Wave II as well as purging at Wave III. Those who purged at Wave II were more likely to have attempted suicide, received psychological counseling, or engaged in delinquent behaviors at Wave I. Regarding the measures of ADHD symptoms, no significant associations were found for the inattentiveness scale; however, participants who purged at Wave II high higher levels of hyperactivity/impulsiveness at Wave I than those who did not. Finally, no significant relationships were found regarding the active coping scale variable.

Of the measures of childhood adversity, having never lived with one (or both) parent (s) and parental poverty were associated with an increased likelihood of purging at Wave II. Not eating with parents, losing a parent to death, childhood neglect, and childhood physical abuse were not significantly associated with purging.

Table 3 shows the multivariable multinomial regression model that included only variables that had a significant relationship with purging at Wave II and/or Wave III. In this model, suicide attempts, body image, depression symptoms, hyperactivity, having psychological counseling, and never having lived with one or both parents were not significantly associated with purging at either wave.

**Table 3:** Multivariable comparison of purging at waves II and III using measures significant at univariable level

**Variable**	**Purging behavior**	**Coefficient**	**Std. error**	**p-value**	**CI**
Weight perception (Likert)	Wave II	0.19	0.47	0.68	-0.74, 1.13
	Wave III	-0.43	0.63	0.49	-1.67, 0.81
	Wave III - Wave II	-0.63	0.74	0.40	-2.09, 0.84
Body mass index	Wave II	0.05	0.04	0.23	-0.03, 0.12
	Wave III	**0.09**	**0.04**	**0.01**	**0.02, 0.16**
	Wave III - Wave II	0.04	0.06	0.45	-0.07, 0.16
Depressive symptoms scale	Wave II	0.07	0.04	0.06	0.00, 0.15
	Wave III	0.09	0.05	0.06	0.00, 0.19
	Wave III - Wave II	0.02	0.06	0.78	-0.11, 0.14
Hyperactive/impulsive scale^3^	Wave II	0.09	0.08	0.25	-0.06, 0.24
	Wave III	-0.11	0.16	0.49	-0.41, 0.20
	Wave III - Wave II	-0.19	0.18	0.29	-0.56, 0.17
Delinquency scale	Wave II	0.17	0.12	0.15	-0.06, 0.41
	Wave III	**-0.89**	**0.37**	**0.02**	**-1.63, -0.15**
	Wave III - Wave II	**-1.07**	**0.38**	**0.01**	**-1.83, -0.31**
Self-esteem scale	Wave II	**-0.18**	**0.07**	**0.01**	**-0.32, -0.04**
	Wave III	-0.10	0.10	0.31	-0.29, 0.09
	Wave III - Wave II	0.08	0.12	0.48	-0.15, 0.31
Had psychological counseling	Wave II	0.73	0.39	0.06	-0.04, 1.49
	Wave III	-0.21	0.91	0.82	-2.00, 1.59
	Wave III - Wave II	-0.93	1.02	0.36	-2.95, 1.09
Attempted suicide	Wave II	-0.04	0.60	0.95	-1.22, 1.15
	Wave III	0.50	0.76	0.51	-1.00, 2.01
	Wave III - Wave II	0.54	0.92	0.56	-1.28, 2.37
Parental poverty	Wave II	**0.44**	**0.21**	**0.04**	**-0.29, -2.35**
	Wave III	-0.17	0.47	0.72	-1.10, 0.75
	Wave III - Wave II	-0.61	0.44	0.17	-1.47, 0.25
Never lived with parent (s)	Wave II	1.03	0.67	0.12	-0.29, 2.35
	Wave III	-0.31	1.17	0.79	-2.63, 2.01
	Wave III - Wave II	-1.34	1.30	0.30	-3.92, 1.23

Note: All risk factor variables were measured at Wave I except those marked ^3^; the latter were measured at Wave III via self-reported retrospective recall. Those reported in bold are significant at p<0.05.

However, parental poverty and self-esteem were associated with an increased likelihood of purging at Wave II. Specifically, those who purged at Wave II had higher levels of parental poverty and lower self-esteem. BMI was not significantly associated with purging at Wave II, but those who purged at Wave III had significantly higher BMI levels at Wave I.

Contrary to what we had hypothesized, it was found that those who purged at Wave III had significantly lower rates of delinquent behavior at Wave I. Additionally, those who purged at Wave III had lower rates of delinquency than those who purged at Wave II.

## Discussion

The overall aim of this study was to examine a broad range of potential adolescent risk factors for developing purging behaviors in a nationally representative sample of U.S. young adult women. Demographic variables did not differentiate risk for the development of purging behaviors. In univariable analyses, several childhood variables were identified to increase risk for purging. Higher BMI, suicide attempts, overweight self-perception, parental poverty, depression symptoms, hyperactivity-impulsivity, delinquent behaviors, low self-esteem, and never having lived with one (or both) parent (s) were associated with purging at Wave II. Higher BMI and depressive symptoms were associated with purging at Wave III. Finally, lower levels of self-esteem were associated with an increased likelihood of purging at Wave II compared to Wave III.

Of the demographic variables controlled for in our study, purging at Wave I was the only variable that was associated with purging at either follow-up time point. Neither participants’ age, race/ethnicity, nor highest level of parental education was found to be associated with purging at Wave II or III. The lack of age differences across the two groups is not particularly surprising given the fact that the Add Health sample has a restricted age range. Consistent with a growing literature that reports that girls or women from various racial/ethnicity backgrounds are equally likely to develop disordered eating, [26,27] our results suggest that purging may be a problem across various racial or ethnic groups. Our finding that highest level of parental education, an indicator often used in the literature as a proxy for parental socioeconomic status, did not differ when comparing purging groups warrants comment in light of our result that family poverty did differentiate the two groups. Both variables were measured using parent/guardian self-report. While parental education does not appear to increase risk for purging, experiencing economic hardship may elevate such risk as will be discussed in more detail below.

Regarding the purging variable, it was found that those who reported purging at Wave I were less likely to also report purging at Wave II or III, and purging at Wave I was not associated with a difference in the likelihood of purging between Wave II and III. It is possible that this inverse relationship is a byproduct of how purging was measured in this study, given that the variable only reflected purging behavior over the last seven days. More frequent longitudinal assessments might better capture the extent to which purging behavior is stable between adolescence and young adulthood.

Additionally, our study confirmed several risk factors for purging that have been shown to predict risk for eating disorders in previous studies, and identified previously understudied time varying effects for several of these variables. For example, self-perception of being overweight significantly predicted purging at Wave II but not at Wave III. Research consistently has found that body image concern (operationalized in a variety of ways in different studies) contributed to increased risk for eating disorder symptoms [2,28] and, therefore, has been targeted as a key risk factor to be addressed in prevention programs [29]. However, our results suggest that this may only be a risk factor over a certain time range. Given the stability of these risk factors over time, it’s surprising that some Wave I predictors that showed significant relationships at Wave II did not predict Wave III purging behaviors. Again, more intensive longitudinal data collection would be useful to more clearly determine how risk factors may affect the likelihood of purging between adolescence and young adulthood.

Similarly, consistent with previous studies, low self-esteem, [28] depression, [28,30,31] and impulsivity [32-34] were significantly associated with increased risk for purging. In addition, we found significant relationships between purging and self-reported suicide attempts as well as between purging and having received psychological counseling. In a series of case control studies, Fairburn and colleagues found that “parental separation” (e.g., due to prolonged illness) and “parental loss” (e.g., due to death) were risk factors for developing an eating disorder [35,36]. In our study, never having lived with one (or both) parent (s) was a risk factor for purging, but having lost a parent to death was not. We cannot answer whether these discrepant findings are a function of methodological differences (e.g., case–control design versus longitudinal design; differences in how the target groups were defined). As noted by Fairburn and colleagues (35), parental separation and loss are also risk factors for other psychiatric problems. Therefore, these parental risk factors may be indirectly associated with the development of eating disorders. For example, parental separation or loss may increase the risk of developing psychiatric problems, which in turn, could increase the likelihood of eating disorders. Future research that examines the nature of the association between these parental factors and eating disorders is important to determine how parental separation and loss might influence eating disorder risk in terms of being mediated or moderated by other risk factors, such as depression. Finally, in our sample, family poverty was found to be predictive of purging onset. This bolsters emerging literature that suggests a relationship between socioeconomic hardship and unhealthy weight control behaviors [37].

Several limitations need to be acknowledged. Although Add Health is a large epidemiological study, the sample still was not large enough to test hypotheses about risk factors for purging in young adult men. Therefore, our findings should not be generalized to boys or men. As is common in large-scale epidemiological studies, key study variables were measured using self-report questionnaires, which tend to be less reliable than interview based measures. Childhood abuse variables as well as the ADHD-IN and -HI scales were collected retrospectively and were, therefore, subject to recall errors. Additionally, because frequency information was not available for the purging behaviors, we were unable to study severity of the behaviors. Interval censoring may also be an issue, given that questions only addressed purging in the last seven days, which did not allow us to identify whether those reporting purging during those days were new onset purgers or whether they may have purged prior to the assessment period. As a result, purging is likely to be underestimated.

Finally, information was not available regarding binge eating behaviors or other extreme weight control behaviors, such as extreme dietary restrictions or excessive exercise. Further studies would be needed to compare our results regarding purging to these similar unhealthy behaviors.

These limitations were offset by several strengths. The study involved a community sample, thus not featuring the limitations inherent in studies of patient samples. The relatively large sample size afforded us the opportunity to use multivariable modeling of risk factors for purging, a relatively uncommon yet clinically important behavior. As well, Add Health collected data on numerous psychosocial variables, allowing us to explore factors that have been considered in psychopathology research regarding other disorders but less so in studies of eating disorders. Finally, our study covered a considerable period of time (8 years) from adolescence into early adulthood.

## Conclusions

This study prospectively examined risk factors for young adult purging in a large nationally representative sample of adolescent females. Results provided support for several risk factors for purging that have been shown to predict risk for eating disorders in previous studies. Future research on the development of purging behavior might include measures of economic adversity and self-esteem to better predict purging in young adults.

## Competing interests

The authors have no conflicts of interest to report.

## Authors’ contributions

EMS conducted all analyses, contributed to methodology selection, and provided data interpretation to the manuscript. JR supervised the design of analyses and contributed to interpretation of data and revisions to drafts of the manuscript. LK contributed to the conception of the study as well as the drafting of the manuscript. FR made significant revisions to the original manuscript for intellectual content. RSW made substantial contributions to the original manuscript and supervised all data analysis and interpretation. All authors read and approved the final manuscript.
